# Rhabdomyosarcoma: a rapidly growing malignancy

**DOI:** 10.11604/pamj.2015.22.121.7479

**Published:** 2015-10-12

**Authors:** Prashanth Panta, David Felix

**Affiliations:** 1 Department of Oral Medicine and Radiology, MNR Dental College and Hospital, Narsapur road, Sangareddy(502294), Medak District, Telangana, India; 2 Postgraduate Dental Dean, NHS Education for Scotland, Westport 102, West Port, Edinburgh EH3 9DN, United Kingdom

**Keywords:** Sarcoma, muscle tumor, striated muscle, childhood malignancy

## Image in medicine

An 8 year old boy presented with a swelling on the left side of the face (A). On inquiry, he revealed that it had been rapidly growing since he first noticed 15 days earlier, and was associated with sharp pain at night. He had constitutional symptoms such as weight loss and fatigue, but no history of fever. On examination, the swelling had a shiny appearance, with no changes in skin color and the mouth opening was partly restricted. There was no local rise of temperature. The left submandibular lymphnodes were palpable, non-tender and fixed. Intraorally, there was a large nodule (B) measuring 5x3 cms, in the lower left buccal sulcus. The nodule was red in color, covered by necrotic slough, and showed areas of bleeding. It was tender, indurated and fixed to the underlying structures. The differential diagnosis included Burkitt's lymphoma, rhabdomyosarcoma, Ewing's sarcoma. On radiographic examination, there were no cystic areas, and bony erosions with respect to the mandible. An incisional biopsy was performed and the microscopic features suggested “Rhabdomyosarcoma-Embryonic type”. The patient was treated with chemotherapy (vincristine, actinomycin-D, cyclophosphamide) plus radiotherapy (5200cGy for 8weeks). No recurrence has occurred during a six month follow up period, and he is still under long term review. Rhabdomyosarcoma is a rapidly growing, childhood malignancy of striated muscle, that exhibits some male predilection. Rhabdomyosarcoma of the oral cavity accounts for 10-15% of all the head and neck cases; the most common sites are tongue, palate and buccal mucosa.

**Figure 1:**
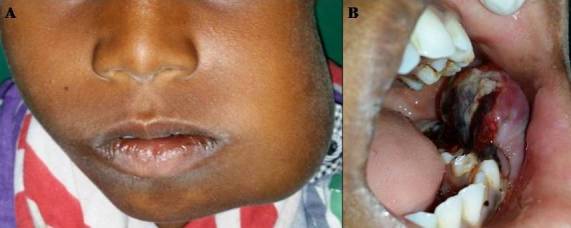
(A) a swelling on the left side of the face; (B) a large nodule measuring 5x3 cms

